# Role of Lifestyle in Neuroplasticity and Neurogenesis in an Aging Brain

**DOI:** 10.7759/cureus.10639

**Published:** 2020-09-24

**Authors:** Reeju Maharjan, Liliana Diaz Bustamante, Kyrillos N Ghattas, Shahbakht Ilyas, Reham Al-Refai, Safeera Khan

**Affiliations:** 1 Neurology, V.N. Karazin Kharkiv National University, Kharkiv, UKR; 2 Neurology, California Institute of Behavioral Neurosciences & Psychology, Fairfield, USA; 3 Family Medicine, California Institute of Behavioral Neurosciences & Psychology, Fairfield, USA; 4 Internal Medicine, California Institute of Behavioral Neurosciences & Psychology, Fairfield, USA; 5 Medicine and Surgery, CMH Lahore Medical College and Institute of Dentistry, Lahore, PAK; 6 Surgery, California Institute of Behavioral Neurosciences & Psychology, Fairfield, USA; 7 Pathology, California Institute of Behavioral Neurosciences & Psychology, Fairfield, USA

**Keywords:** neurogenesis and lifestyle, neurogenesis and neuroplasticity and lifestyle, neurogenesis and neuroplasticity, neuroplasticity and lifestyle

## Abstract

Neuroplasticity is the brain's ability to transform its shape, adapt, and develop a new neuronal connection provided with a new stimulus. The stronger the electrical stimulation, the robust is the transformation. Neurogenesis is a complex process when the new neuronal blast cells present in the dentate gyrus divide in the hippocampus. We collected articles from the past 11 years for review, using the Medical Subject Headings (MeSH) strategy from PubMed. Quality appraisal was done for each research article using various assessment tools. A total of 24 articles were chosen, applying all the mentioned inclusion and exclusion criteria and reviewed. The reviewed studies emphasized that modifiable lifestyle factors such as diet and exercise should be implemented as an intervention in the elderly for healthy aging of the brain, as the world's aging population is going to be increased, leading to the expansion of health care and cost. Multiple studies have publicized the relation of diet and exercise with cognition function in aging people. A diet consisting of curcumin in its food has its anti-oxidative property, which prevents rapid aging of the brain, other diet patterns such as a caloric restriction diet can influence brain plasticity and preclude the decline of memory. Exercise can increase brain-derived growth factor (BDGF), vascular endothelial growth factor (VEGF), synapsin one, and tyrosine kinase activity that can expand the size of the brain, enhance the plasticity and neurogenesis. This review aimed at exploring lifestyle factors that contribute to neuroplasticity and neurogenesis. Thus, providing a new path for clinicians and researchers to map out the future possible significant benefits for optimal brain aging in a healthy fashion.

## Introduction and background

A concept in neurobiology states that 'neurons that fire together, wire together' suggesting that the number of particular neurons that get stimulated during an experience, there is the maintenance of synapses and those neurons get strongly connected with each repetitive specific stimulus [1]. There is going to be an increase in the aging population, which will lead to a concomitant growth in age-related cognitive decline (ARCD). Approximately 8.5% of the world's population is aged >65 years (~617 million) and is expected to be 16.7 % by 2050 (~1.6 billion) leading to the personal, social, and economic burden of care for an individual with age-related problems which can delay healthy lifestyle measures [2,3].

The plasticity of the brain is defined as the brain's capacity to modify and shape itself into a different form, ability to act, and adapt in a certain way with the given experiences. Due to the brain's plasticity to transform as per the environment's need, each new experience acts as a protective reserve for that individual inclined to age-related brain transformations and pathology [2-4]. Neurogenesis is a complicated process in which the stem cells of neurons present in the hippocampus region of the brain differentiate and proliferate into new neurons with new experiences and other supporting cells. This process is affected by many intrinsic and extrinsic factors [5,6,7]. Aging, neuroinflammation, oxidative stress, and brain injury are factors that may affect neurogenesis. Adult neurogenesis is negatively affected by high fat and high sugar diet, alcohol, and opioid addiction [5]. Globally, at the rate of every one in seven persons are detected with dementia [5]. A successful minimal delay of the onset of dementia can reduce age-related neurodegenerative disease, which makes it one of the rising problems in this world and opening the doors for a new level of scientific research in this area for the solution [2,6]. The brain functions can modify with lifestyle changes. Certain modifiable factors like diet, stress, exercise can have a positive impact on the brain's cognitive reserve, which means the brain's ability to sustain its normal function during aging can threaten with neurodegenerative disease, injury, and aging. One of the most important reserves is present in the hippocampus region of the brain that makes the brain more plastic and allows it to be adaptive in any situation of cognitive demand. As the situation demands a more complex and creative response, the brain starts to act in that dimension as the demand of the respective environment [7].

Lifestyle factors have an immense impact on predicting in decline rate of cognitive functions and skill, leading to age-related neurodegenerative diseases like Alzheimer's disease (AD). Women who were physically active in their teens showed a significantly lower possibility of cognitive impairment in life. Yaffe et al. conducted one of the significant prospective studies in which the cognitive decline of an older adult population was measured at its baseline. This study showed that 30% of the participants maintained cognitive function, 53% showed a mild decline, and 16% has severe cognitive decline [6]​​​​​​**. **This experiment highlighted that if one of the preventive measures is implemented before the declining function, significant cognitive decline can delay for some period. Also, Exercise and increase consumption of polyphenols can intensify brain functioning [8].

A study was done in male Long-Evans rats in which they were allowed to follow either a regular diet or a diet with elevated levels of saturated fats, and were studied in variable-interval delayed alternation task [9]. The impairment to learn basic alternation rule and their ability to remember trial-specific information over time was significantly reduced in the rats who were in an elevated level of saturated fats than who was on a balanced diet. The rats who were on a higher level of saturated fat and cholesterol had more working memory errors in the water radial arm maze. The error was even more significant when they were intellectually challenged with high memory loads. The experiment demonstrated the effect of such fast food on cognition, and that it causes a relatively more sudden negative impact on cognitive skills [9].

There is undoubtedly a beneficial impact of exercise and diet in developing neurogenesis. However, the mechanism of neurogenesis and plasticity is not well understood. Its pathogenesis related to the development of the age-related neurodegenerative disease is not clear and is yet to be explored [10]. There is a distinct relationship between brain plasticity and healthy lifestyle modification. Due to the dynamic relationship and the overbearing impact of an active lifestyle in neurogenesis in promoting healthy aging and delaying the aging progress, several national bodies like the National Institutes of Health, Centers for Disease Control, the Alzheimer's Association, and the American Association of Retired Persons (AARP) have come forward to justify its importance [10]. Diet and exercise can harness neurogenesis and optimal brain health level, delay the onset of the aging process, highlight implications for clinicians and researchers [2].

The primary motive of this article is to illuminate the significant difference in the cognitive function of the brain after healthy lifestyle changes. This article focuses mainly on physical exercise and diet, as well as the development of neurogenesis and neuroplasticity.

## Review

Method

Eligibility criteria: Articles were independently checked for eligibility criteria. We chose the articles that mainly focused on exercise and diet as lifestyle modification and excluded other risk factors such as alcohol, smoking, high blood pressure. Studies from the last eleven years were selected. We only included the papers written in English and excluded the ones written in other languages. The included articles focused on the interventions discussed in both humans and animals.

Information sources: We used PubMed as our main database to collect our data. Articles were retrieved from 2009 to June 6, 2020. The keywords used were "Neurogenesis", "Neuroplasticity", "Exercise", "Diet", "Lifestyle modification", and "Brain".

Study selection: A comprehensive computerized search was conducted using the Medical Subject Headings (Mesh) strategy to identify the relevant articles. The titles and abstracts were screened in detail to evaluate their relevance, to be chosen for this review. We identified a total of 35 articles after the initial screening process. Duplicate studies were removed and inclusion and exclusion criteria were applied. Twenty- four articles were finalized for the review. The risk of bias was minimized by using the relevant quality assessment tools for different types of articles.

Results

A total of 24 articles were finalized based on their relevance to the topic for review after conducting the quality appraisal of the selected studies. "Neuroplasticity", "Neurogenesis", "Brain" and "Lifestyle", keywords were used separately or in combination to get the articles (Table 1).

**Table 1:** Keyword Search Results

Keywords	PubMed
Neurogenesis	20,909
Neuroplasticity	59,322
Lifestyle	176,780
Brain	1,980,739
Neuroplasticity and neurogenesis	3101
Neuroplasticity and lifestyle	233
Neurogenesis and lifestyle	116
Neurogenesis & Neuroplasticity & lifestyle	25
Neurogenesis &Neuroplasticity & lifestyle &Brain with mesh strategy	25

**Figure 1:**
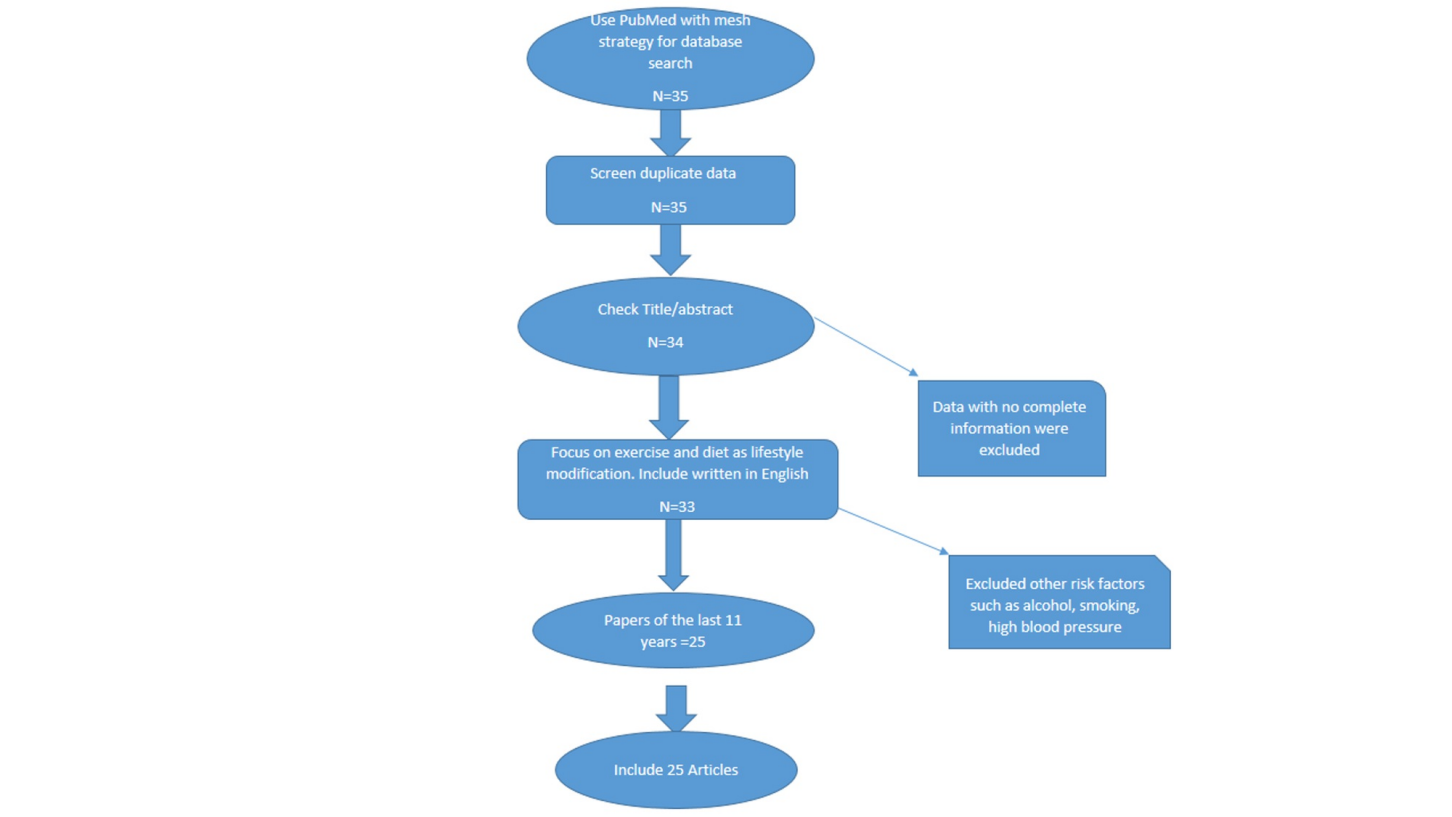
PRISMA Flow Diagram PRISMA: Preferred Reporting Items for Systematic Reviews and Meta-Analyses

The data were screened and the total number of articles were identified during the data extraction using a Preferred Reporting Items for Systematic Reviews and Meta-Analyses (PRISMA) flow diagram (Figure 1) [11].

Discussion

Lifestyle plays a pivotal role in mental and physical well-being. Diet and exercise are essential components to make one healthy. Various studies revealed in modern science about the advantages occurring in the brain, delaying and preventing the aging process.

Effect of Diet on Brain Function and Cognition

Diet is one of the essential factors that can cause the brain to function and structure differently. The way people consume their food can affect their brain positively or negatively depending on the types of food they eat. The brain is one of the vital organs which is 2% of body weight but consumes the most energy, which is 20% of the total expenditure of energy, and this is what drives people to consume their food differently and develop an eating behavior [1]. The intrinsic factors that are least likely to be modified in the process of aging are neuroinflammation, oxidative stress, and brain injury and other factors such as lifestyle that can be modified for slowing the aging process is a low intake of fat and sugar diets and alcohol and avoid opioid addictions [2]. One of the diet modifications can be done by adding polyphenols (e.g., phenolic acids, stilbenes, lignans, flavonols, and anthocyanins) which consists of 800 compounds with an antioxidant property that can be found in fruits, vegetables, tea, wine, juice, plants, and some herbs [3]. Even though polyphenols are not considered essential nutrients, these compounds can decrease the risk of age-related cognitive problems. The food which contains an abundant number of macronutrients like omega-3 fatty acids omega-6 poly-unsaturated fatty acids and flavonoids are found in vegetables, fish, and nuts that develop a favorable environment for the brain to generate new connections and synapsis and make that synapsis strong [1-4]. Polyphenols play vital roles in neuroplasticity, neurogenic, anti-inflammatory, and neuroprotective effects, especially this effect is observed with curcumin, catechins, resveratrol, and omega-3 fatty acids [4].

Curcumin: Curcumin is a plant-based diarylheptanoid that produces yellow pigment mostly used in curries as it can modulate various signaling properties because of its polyphenol properties. It has anti-inflammatory, anti-bacterial, cytotoxic, antioxidant proliferative, wound healing, and anti-nociceptive properties [12,13].

Curcumin’s anti-inflammatory properties can be modulated by pro-inflammatory cytokines. It is activated by pro-inflammatory transcription factors nuclear factor kappa-light-chain-enhancer(NF-κB) the pro-inflammatory transcription factors NF-κB and signal transducer and activator of transcription three mediates inflammatory response [12]. Curcumin has antioxidant and free-radical properties, which have been shown in various studies. The antioxidant property of curcumin is due to the hydroxyl group or methylene group of the β-diketone (heptadiene-dione) moiety [12]. The phenolic hydroxyl group of curcumin has antioxidant activity that is produced endogenously including superoxide dismutase (SOD), catalases, peroxiredoxins, and glutathione, which play critical roles in preventing endogenous and xenobiotic-induced oxidative damage [13]. Also in another study, it states anti-oxidant therapy is not enough to prevent age-related neurodegenerative disorder. They mentioned the removal of oxidative deterioration by scavenging oxidants [14]. A modern drug has been designed for an age-related neurodegenerative disease, which includes an active ingredient of curcumin derivatives that exerts the inhibitory effect both tau and amyloid-β (Aβ) aggregation. Curcumin can bind in amyloid-beta protein, it's fluorescent property can help in early detection of Alzheimer's disease [15]. The development of the more powerful aggregation inhibitor 3-[(1E)-2-(1H-indol-6-yl) ethenyl]-5-[(1E)-2-[2-methoxy-4-(2-pyridylmethoxy) phenyl] ethenyl]-1H-pyrazole (compound four) has a better pharmacokinetic profile and pharmacological efficacy in vivo than curcumin, making it also suitable for the age-related degenerative disease [16].

Curcumin is a polyphenol that is one of the chief spices of Asian cuisine. The benefit of curcumin has been examined extensively, which shows the potential benefit of healthy brain growth and also the process of neurogenesis [17]. Aged Sprague-Dawley rats (age 15 months; male) were given a 0.048% curcumin diet or unpurified diet for six or twelve weeks. Rats showed improved performance on an olfactory cortex-based social recognition memory task at both six and twelve weeks, and on hippocampus-dependent spatial learning and memory task after 12 weeks, relative to controls. The rats that were administered curcumin showed increased proliferation in the dentate gyrus (DG) at 12 weeks, relative to diet controls, which show the process of neurogenesis in the olfactory cortex [18]. The vital role of neurogenesis has been played not only in animals but also in humans. The association between curry consumption and cognitive function is studied in a large group of the elderly non-demented population. The study revealed those population which used curcumin in their diet frequently scored finding that persons who frequently consumed curry scored meaningfully well on the Mini-Mental State Examination comparatively to those who irregularly consumed curry. The next study of a six-month randomized, placebo-controlled, double-blind, clinical study of curcumin in persons with progressive cognitive decline and memory found elevated serum amyloid beta-40, but not improvements on the Mini-Mental State Examination [17].

Various studies provide the evidence in its neurogenesis with curcumin; however, none of the studies precisely identified as what causes a person to maintain a neuronal growth with the use of this spice. The chief drawback of curcumin to be used as medicine is its poor bioavailability. If the scientist and researcher can modify curcumin in such a way that a vast amount is absorbed, then it would be more straightforward to assess the effect of this yellow pigment. More studies are still required to be conducted to identify its effect on brain growth and soon will be able to use this ingredient in the medicine to prevent neurodegenerative disease.

Dietary Energy Restriction: The hypothalamus of the brain regulates a dietary intake (calorie); that is why it is believed to deliver a significant impact in the brain by the diet we consume. Multiple studies have been undertaken in rats and mice, and the evidence from them shows sustained excessive energy intake can adversely affect the cognitive function of the brain. Excessive energy intake can result to decrease cognitive function, increase the risk of age-related disorders. It makes neurons vulnerable to aging, and neurodegenerative disorders such as Alzheimer's disease and dietary restriction can enhance plasticity and decrease the rate of memory loss and enhance the capacity of stress response of the brain [17]. However, the benefits are noticed with adherence to quality diet; maximum improvement in cognitive decline with higher adherence to a more nutritious diet [18]. Also, Nuturis® (Nutricia), an infant milk formula, when introduced in the early life can change the harmful effects of Western-style diet in adulthood, in hippocampal neurogenesis [19]. Nuturis® (Nutricia), which contains physical characteristics of human milk (lipid globules), can be targeted at early life to affect metabolic and mental health in adulthood [19].

Effect of Exercise on Brain Function

Neuroprotection: The prefrontal lobe is responsible for working memory [20]. Chapman et al. study of healthy sedentary individuals with shorter duration of exercise found (three months) had gained in hippocampal size and blood flow hence increasing memory performance [21].

Executive function and temporal lobe are responsible for long term memory [22]. In the animal studies, the effects were seen at molecular levels such as neurogenesis, synaptogenesis, gliogenesis, and angiogenesis. It occurred due to the increase expression of neurotransmitter and neurotrophin [23]. Lifestyle not only plays a role directly in neurogenesis but also stimulates microglia. The microglia (protector of neuronal cells) indirectly facilitates neurogenesis [7]. The experiment conducted in mouse conducted showed it is possible to delay the cognitive decline only if a healthier environment is maintained, such as physical activity and diet. If not maintained, then the benefits remain inconclusive [24]. They are likely to be increased during physical exercise. These cortices are most vulnerable to aging and also severely affected due to Alzheimer's disease (AD) [25, 26]. In the study conducted in 2011, the authors concluded the incorporation of aerobic exercise in daily life at adulthood can lead to the increment of hippocampal size (by 2%), eventually, reverse the age-related volume reduction of the hippocampus by one or two years [27]. Chronic stress can cause severe damage in the hippocampus, thus leading to a substantial decrement in neurogenesis. Hippocampal structural plasticity and neurogenesis can be modulated by hormones like sex, stress, and metabolic [28]. Physical exercise mostly increases the anterior part of the hippocampus that is responsible for spatial memory performance [29]. There is a tight relationship between the exercise and angiogenesis. The blood volume increased with an increase in the intensity of the exercise by the process of angiogenesis (the growth of new blood vessels) [4]. The studies conducted in knocked out mice showed activation of the BDGF tyrosine kinase B(TrkB) pathways during chronic exercise and high interval intensity workout. This pathway is responsible for neuroprotection, neurogenesis, and neuroplasticity [30]. Our brain function and structure adapt according to the cognitive demand of exercise, which then prevents the brain from aging and age-related neurological disease [31]. Brain-derived growth factor (BDGF), vascular endothelial growth factor (VEGF), and insulin-like growth factor (IGF-1) have a direct association with physical activity (PA). As the intensity of PA increases, the expression of these chemicals increases. BDGF is responsible for hippocampal neurogenesis, dendritic complexity, and synaptic complexity and neuroplasticity [20]. Various studies conducted in humans regarding physical activity (PA) and the release of BDGF in the population that did PA have a high level of BDGF compared to those individuals that did not. BDGF concentration directly correlated with hippocampal volume [20]. An AD patient has a decreased level of BDGF compare to a healthy individual. Vascular endothelial growth factor (VEGF) is responsible for forming blood vessels in the brain, responsible for the process of neurogenesis. The study by Fabel et al. showed that peripheral VEGF is vital for neurogenesis. Inhibition of peripheral VEGF indicated that the exercise-induced neurogenesis was not present [32].

**Figure 2:**
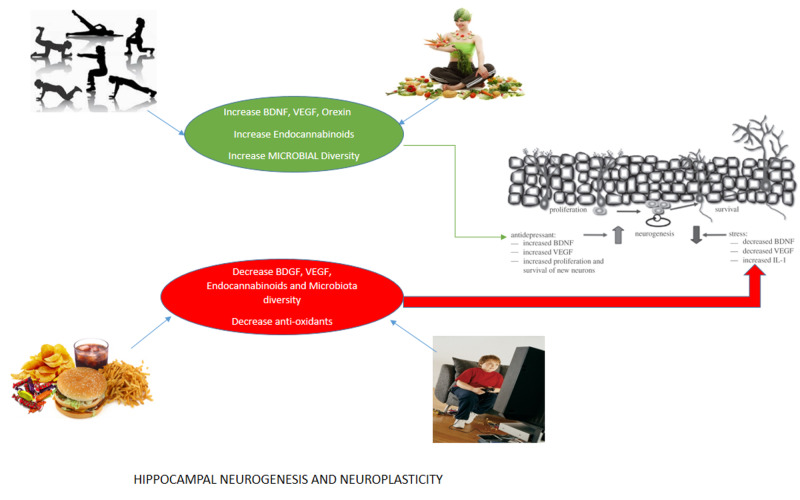
Effects of diet and exercise in the hippocampal neurogenesis and neuroplasticity VGEF: vascular endothelial growth factor; BDNF: brain-derived neurotrophic factors; BDGF: brain-derived growth factor; IL: interleukin

Orexin A and orexin B are neuropeptides synthesized from the lateral hypothalamus. They are accountable for regulating wakefulness and arousal, motivation and emotions, and motor and autonomic functions [33, 34]. Healthy lifestyle adherence can build up brainpower and can lead to more prolonged survival, delayed aging, physical, and mental health [6]. When new neurons are integrated with existing circuits of new and old neurons, some of the old neurons get replaced. Thus, it shows blocking new synapse can prevent the removal of memories from the hippocampus. A naïve connection made by new neurons is flexible, allowing them to learn new experiences and then get integrated into the various network [35]. Figure 2 presents the different neurochemical productions that occur in our body with diet and exercise [30].

Mechanism of exercise-induced neurogenesis: Many researchers have discovered the significant effect of exercise-induced neurogenesis. In one of the studies, male rats forced implementing a treadmill during their adolescence, demonstrated the decrease expression of BDGF throughout the brain. In contrast to this, the voluntary treadmill increases the expression of cannabinoid. It is responsible for hippocampal neurogenesis, especially the proliferation and migration of neurons in the dentate gyrus of the hippocampus [35].

During exercise, cathepsin B (CTSB) releases, and its expression gets enhanced, which results in enhancement of spatial memory, inhibitory transmission onto dentate gyrus of the hippocampus, and decreased hippocampus P11, a protein needed for CTSB effects on neuronal differentiation and migration. In vivo, exercise elevates CTSB plasma level and hippocampal CTSB gene expression, thus enhancing brain function in contrast to when CTSB gets inhibited, thus decreasing spatial memory [36]. A chronic behavior of physical activity up-regulates synaptic plasticity and neurogenesis that can be explained by the Wnt pathway. On the one-hand, adult rats who were on long-term exercise expressed a higher level of Wnt protein, on the other hand, rats those who had a sedentary lifestyle secreted more wnt antagonist, Dkk-1, the lowest level found in the exercised group [10]. Anti-apoptotic proteins such as Bcl-2 increased in mice who did exercise [10].

The elderly who did aerobic exercise responsible for up to 60 min can improve information processing, prevent atrophy of the hippocampus, and increase the neuronal volume of the hippocampus [21]. The most common behavioral changes in the elderly are cognitive loss and behavioral changes related to the atrophy of the brain. They can be delayed by physical exercise, shown in a study performed in the white and black elder population [6].

In the population who completed a moderate amount of physical activity increased BDGF, synapsin facilitates neurotransmitter release; those who consume a high-fat diet have a low level of synapsin-1. Exercise leads to the activation of various pathways mitogen-activated protein kinase (MAPK) such as increase protein transcription and eventually accelerates neurotransmitter release. Hippocampal neurogenesis causes an increase in brain size and a decrease in apoptosis in the hippocampus to prevent in atrophy of the brain, therefore leading to significant prevention of spatial memory deficit in people who do exercise. One of the prime functions of exercise is to decrease pro-inflammatory cytokines, decrease insulin resistance, increase brain insulin signaling, principal to increase protein transcription, eventually expediting neurotransmitter release for spatial memory [37].

In one of the studies, adult hippocampus neurogenesis alone was not enough for improving cognition. Neurogenesis produced minimal benefit, as shown in mice. Reduction of Ab load and increment of BDGF, interleukin-6, fibronectin type III domain-containing protein, and synaptic markers were equally significant along with exercise-induced neurogenesis [24]. Intrinsic (hormones) and extrinsic factors (diet and exercise) are responsible for neuroplasticity and neurogenesis. Based on an exercise regime, duration, and intensity can transform how neuronal plasticity can happen in the brain that can affect therapeutic approaches and help to regain the strength of an aging brain [5]. One of the forms of exercise is an acrobatic exercise that involves multiple muscle groups and produces a compound movement that can not only heightened neurogenesis but also promote learning. The study conducted in rats, which then evaluated includes the study of the cerebellum, the motor cortex, striatum, and hippocampus. The result of this study concluded, exercise stimulates plasticity in brain areas of rats that are chiefly affected by the intensity and duration of the PA [28].

BDGF increased in production and secretion during the exercise. It causes an increase in messenger ribonucleic acid (mRNA) production expression of its receptor, tyrosine kinase. The function of BDGF decreased when the tyrosine activities are blocked. Thus, BDGF functions: neuroplasticity, and neurogenesis are drastically reduced [30]. Newly formed neurons help on learning, memory function, and integration into the hippocampal network circuit. These new neurons present in the hippocampus possess a diverse kind of role, at a different stage that helps to form pattern separation and memory formation. Integrated neurons and newly formed neurons aid in learning function [30]. Moreover, the enriched environment can nourish an aging brain, improving the brain function at its molecular level ( increasing new blood vessels, synapsis, glial cells, and neuronal cells) thus preventing a rapid decline in cognitive function [23].

In one of the studies, different types of exercises were compared. Running and aerobic exercise causes adult hippocampal neurogenesis (AHN) to resistance training. With the same intensity and time duration of exercise, resistance training did not increase in BDGF in the long run, whereas running and aerobic exercise significantly improved BDGF [38]. The effectiveness of volunteered exercise compared between adolescent and adult-onset. The result of the study showed adolescent-onset exercise tipped toward increased AHN and enhanced neurogenesis, compare to adult-onset exercise [39]. Neuroplasticity is modulated by exercise intensity, interval, and duration, thus increasing BDGF, responsible for immature neuroblasts to proliferate and to increase its survival [40, 41]. The level of activity in a treadmill also improves learning, memory, and long-term potentiation, according to the study performed in mice [42]. The summary of the studies reviewed for this article is presented in Table 2.

**Table 2:** The total number of reviewed articles CR: caloric restriction; PUFAs: polyunsaturated fatty acids; WSD: Western-style diet; BDGF: brain-derived growth factor; PA: physical activity;

AUTHOR	Year of publication	Purpose of the study	Result/ Conclusion
Philips C et al. [2]	2017	Importance of lifestyle modification for protecting cognitive function and brain health during aging.	Neuroprotective, neuroplastic, neurogenic, inflammatory effects by diets modification especially curcumin, catechins, resveratrol, and omega-3 fatty acids. It showed a straight relation between increased levels of PA and improved cognition, with increased hippocampal volume seen after chronic exercise.
Poulose SM et al. [5]	2017	To study factors affecting neurogenesis.	Folates, vitamin E, Omega-3 fatty acids, and polyphenols found to mitigate aging and age-related behavioral declines.
Rajaram S et al. [3]	2019	To gain proofs for vegetarian diets prevent the brain aging process	Consumption of 100% fruit juices, berries, cocoa, coffee, green tea, and nuts to improve cognitive function among elderly adults.
Murphy T et al. [18]	2014	To study the effects in neurogenesis with calorie restriction (CR), intermittent fasting, polyphenols, and poly-unsaturated fatty acids (PUFAs)	A multitude of dietary regimens and components increase the levels of adult hippocampal neurogenesis and neurotrophins as well as acting to enhance the synaptic function.
Abbink MR et al. [19]	2020	To investigate Nuturis®(Nutricia) could modulate age-induced alterations in later life.	Early-life exposure to a diet containing Nuturis®, better mimicking some of the chemical structure of lipid globules in mammalian milk. It can modulate the hippocampal response to adult Western-style diet (WSD) at a structural level.
Gelfo F et al. [23]	2018	To gain knowledge about experiences can change brain function.	The transformation of brain health and function can be caused by experiences.
Valero J et al. [7]	2009	To study how microglia leads to neurogenesis.	The hippocampal neurogenesis and microglia connection is modulated by lifestyle choices, maintains brain reserve.
Choi SH et al. [24]	2018	To study the effects of exercise and BDGF.	Elevated BDGF can induce neurogenesis.
Paredes JT et al. [28]	2016	To study how hormones and exercise make a difference in neuroplasticity.	The change of hippocampus neurogenesis based on duration, intensity, and frequency of exercise.
Hueston CM et al. [29]	2017	To study the long term effects of the central nervous system disturbances.	Stress can lead to long term detrimental effects.
Raichlen DA et al. [31]	2017	To study how physical activity transforms the brain function and structure.	Exercise can change the structure and function during the aging process.
Raefsky SM et al. [1]	2017	To explore how the adaptive neuronal response can lead to optimal brain function.	Difficult circumstances, physical activity, and fasting can prevent early aging.
Praag HV et al. [8]	2009	To study how physical activity benefits learning, memory, and cognitive function.	Diet and exercise have synergy in between them and activates the same pathway for neurogenesis
Chieffi S et al. [4]	2017	To access how physical activity can delay aging and its associated neurodegenerative disease.	Physical activity stimulates bio mechanisms that lead to delay in the brain aging process.
Mattson M et al. [9]	2010	To study the increased intake of diet and insulin resistance on brain function.	The cellular stress response triggers during caloric restriction and leads to adaptive changes in the brain.
Bayod S et al. [10]	2014	How WNT pathway activation can cause neurogenesis.	Neuroprotection is present in the activated Wnt pathway.
Yaffe K et al. [6]	2009	To know how to maintain the cognitive function of elderly	Healthy lifestyle adherence maintain the mental health of elderly
Alam MJ et al. [35]	2018	To study how neurogenesis can preserve hippocampus memory capacity.	A new experience can renew hippocampal circuits and maintain hippocampal memory.
Moon HY et al. [36]	2016	To find out running can release cathepsin B and muscle secretory factors that help in cognitive function.	Memory and neurogenesis were not affected by neurogenesis. However, it enhances the process of adult neurogenesis.
Loprinzi PD et al. [37]	2019	To know whether a high-fat diet can be compensated with exercise.	Exercise can counteract the effect of high fatty food.
Nokia MS et al. [38]	2016	How aerobic exercise can cause adult hippocampal neurogenesis.	Adolescents and adulthood exercise affect differently in the hippocampus.
O’Leary JD et al. [39]	2019	To find out how both exercise and diet can affect chronically stressed mice.	Increase in several immature neurons in the hippocampus with diet and exercise in chronically stressed mice.
Hutton CP et al. [40]	2015	To find out the collective effect of diet and exercise on the hippocampal function of mice those who is stress for a long time.	The combination of dietary supplementation and exercise had multiple beneficial effects, as shown in multiple areas of the brain and as well as increased signaling process in the brain.
Gutierrez RMS et al. [41]	2018	How acrobatic exercise can cause neuroplasticity.	Acrobatic exercise can impact neurogenesis depending on intensity, duration, and frequency.

## Conclusions

This review contains various studies that provide evidence about lifestyle (diet and exercise) and its association in neurogenesis. Curcumin remains an ingredient that can cause a substantial effect on neuroplasticity. Dietary patterns and excessive diet restriction can hurt preventing age-related neurodegenerative diseases. In the same way, physical exercise can exert an ample effect on the neural aging process. PA causes to release various chemicals such as BDGF, VEGF, orexin that plays a sizeable difference not only in the process of angiogenesis but also the growth of new neural cells in the dentate gyrus in the hippocampus.. Age-related pathologies, memory loss, decrease in learning capacity increase as the number of the elderly population grows. It causes a direct increase in the cost of health care and quality of life. Based on the review the studies available are predominantly in animals and fewer studies conducted in humans. The studies in two distinct species do provide a positive result. More studies should be conducted in humans to show significant neurogenesis and neuroplasticity. Such that it will allow doctors to employ it as an intervention in their clinical practice. Therefore, more research should be conducted in humans to explore more advantages and also for the prevention of rapidly declining age-related disorders.
